# Fibrillar pharmacology of functionalized nanocellulose

**DOI:** 10.1038/s41598-020-79592-5

**Published:** 2021-01-08

**Authors:** Sam Wong, Simone Alidori, Barbara P. Mello, Bryan Aristega Almeida, David Ulmert, Matthew B. Brendel, David A. Scheinberg, Michael R. McDevitt

**Affiliations:** 1 Department of Radiology, Memorial Sloan Kettering Cancer Center, 1275 York Avenue, Box 231, New York, NY 10065 USA; 2 Department of Chemistry, Hunter College, New York, NY 10065 USA; 3 Molecular Pharmacology Program, Memorial Sloan Kettering Cancer Center, New York, NY 10065 USA; 4 Molecular Cytology Core Facility, Memorial Sloan Kettering Cancer Center, New York, NY 10065 USA; 5 Department of Pharmacology, Weill Cornell Medicine, New York, NY 10065 USA; 6 Department of Radiology, Weill Cornell Medical College, New York, NY 10065 USA

**Keywords:** Drug discovery, Chemical biology

## Abstract

Cellulose nanocrystals (CNC) are linear organic nanomaterials derived from an abundant naturally occurring biopolymer resource. Strategic modification of the primary and secondary hydroxyl groups on the CNC introduces amine and iodine group substitution, respectively. The amine groups (0.285 mmol of amine per gram of functionalized CNC (fCNC)) are further reacted with radiometal loaded-chelates or fluorescent dyes as tracers to evaluate the pharmacokinetic profile of the fCNC in vivo. In this way, these nanoscale macromolecules can be covalently functionalized and yield water-soluble and biocompatible fibrillar nanoplatforms for gene, drug and radionuclide delivery in vivo. Transmission electron microscopy of fCNC reveals a length of 162.4 ± 16.3 nm, diameter of 11.2 ± 1.52 nm and aspect ratio of 16.4 ± 1.94 per particle (mean ± SEM) and is confirmed using atomic force microscopy. Size exclusion chromatography of macromolecular fCNC describes a fibrillar molecular behavior as evidenced by retention times typical of late eluting small molecules and functionalized carbon nanotubes. In vivo, greater than 50% of intravenously injected radiolabeled fCNC is excreted in the urine within 1 h post administration and is consistent with the pharmacological profile observed for other rigid, high aspect ratio macromolecules. Tissue distribution of fCNC shows accumulation in kidneys, liver, and spleen (14.6 ± 6.0; 6.1 ± 2.6; and 7.7 ± 1.4% of the injected activity per gram of tissue, respectively) at 72 h post-administration. Confocal fluorescence microscopy reveals cell-specific accumulation in these target tissue sinks. In summary, our findings suggest that functionalized nanocellulose can be used as a potential drug delivery platform for the kidneys.

## Introduction

Cellulose is a high molecular weight polysaccharide consisting of a linear chain of β-1,4-linked D-glucose (C_6_H_10_O_5_)_n_ units. Polymeric macromolecules comprised of up to 10^4^ glucose units result in a linear, water-insoluble, durable material. Significant research effort has been dedicated to development of natural polymers^1^ as medical implants, drug delivery excipients, antimicrobials, wound dressings, vascular grafts, and scaffolds for tissue engineering^2^. Highly crystalline regions of cellulose microfibrils can be extracted from bulk cellulose via chemical, mechanical or enzymatic processes eliminating the non-cellulose and amorphous cellulose components yielding cellulose nanocrystals (CNC)^3–10^. These nanomaterials are rigid, rod-like particles consisting of cellulose chain segments organized into crystalline structures. Compared to bulk cellulose, which has a greater fraction of amorphous cellulose, CNC have a high aspect ratio (length-to-diameter ratio of 10–100) and exhibit a high Young’s modulus (10^2^ GPa), as well as a high surface area (10^2^ to 10^3^ m^2^g^−1^), and unique liquid crystalline properties. Interest in the bioengineering and food science applications of CNC is on the increase and there is a need to develop water-soluble functionalized platforms for drug delivery schemes^3–10^.

Fibrillar macromolecules that are rigid and have high-aspect ratios exhibit unique pharmacological properties in vivo compared to high molecular weight globular molecules^11,12^. Our lab has been investigating functionalized carbon nanotubes (fCNT) as synthetic molecular platforms for gene and drug delivery^13–20^. Several physicochemical properties are common to CNT and CNC including high surface area, rigid fibrillar shape, high aspect ratio and a minimal aqueous solubility of the unfunctionalized starting materials. Surface modification of CNT with multiple copies of primary amines provided a means to greatly improve water-solubility and biocompatibility as well as an expedient means to engineer a drug excipient for loading and transport of pharmacologically active cargo. The exceptional pharmacokinetic behavior of functionalized CNT in rodents and nonhuman primates is best described by rapid blood clearance, cell specific-accumulation in kidney and liver and intact renal elimination in the urine^13–15,18^.

Chemical surface modification of pristine CNC is hypothesized to render them more useful as drug and gene delivery vehicles in vivo and encouraged our interest in these safe and biocompatible natural biopolymers. Herein, we describe a first-generation covalent chemical design approach for pristine CNC that improves aqueous solubility and adds functionality in vivo. Further, we report for the first time, the pharmacokinetic profile of radionuclide and fluorescent tracer-labeled of functionalized CNC (fCNC) in vivo.

## Materials and methods

### Chemical modification of raw cellulose nanocrystals

**Figure 1:**
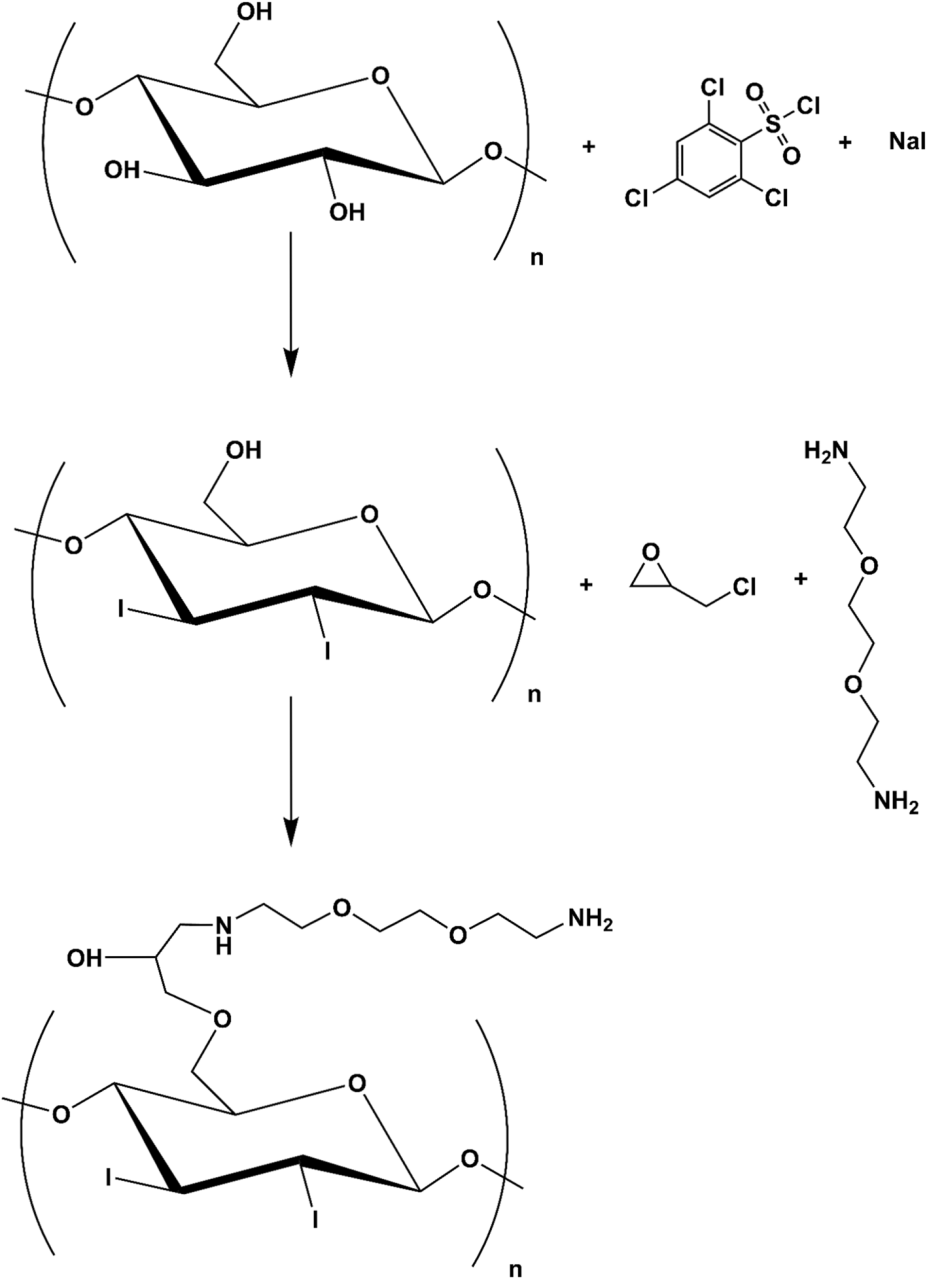
Chemical modification scheme of pristine CNC to yield iodo- and ammonium-functionalized fCNC.

Synthesis of ammonium-functionalized cellulose nanocrystals (fCNC) was carried-out in a two-step process. The first step entails iodination of the glucose secondary alcohol groups (Fig. 1). Pristine technical grade CNC (Blue Goose Biorefineries, Inc., Saskatoon, Saskatchewan, Canada) was suspended in water (7.4% wt./wt.) and taken to dryness under reduced pressure. The resulting white crystalline solid (0.46 g) was suspended in 4 mL of anhydrous pyridine (Sigma Aldrich, St. Louis, MO) and chilled to -15 °C under argon. To the white CNC slurry was added 1 g of 2,4,6-trichloro-benzenesulphonyl chloride (Sigma Aldrich) dissolved in 3 mL of anhydrous pyridine. The reaction mixture was stirred for 90 min at -15 °C and then slowly raised to ambient temperature over 60 min. A 0.5 mL volume of N,N-dimethylformamide (N,N-DMF, Sigma Aldrich) was slowly added to the reaction mixture at ambient temperature and yellow-orange colored precipitate was observed. After 15 min., 1.25 g of NaI (Sigma Aldrich) was added to the reaction mixture that immediately turned brown and was further stirred for 90 min. at 40 °C. The resulting solution was filtered and the white precipitate (I-CNC) was washed extensively with methanol and then dried in vacuo; 0.29 g of I-CNC was recovered. In the second step, I-CNC (0.15 g) was suspended in deionized water with sonication (5 min at 50 W) to disperse the solid. The slurry was then adjusted to pH 11 with the addition of 0.1 M NaOH and epichlorohydrin (Sigma Aldrich) was added in three 0.16 g aliquots; the reaction mixture was stirred for 20 h at 60 °C. A twofold molar excess of 2,2′-(ethylenedioxy)bis(ethylamine) (EBEA, Sigma Aldrich) was then added to react with the reactive epoxide appended onto the I-CNC. The resultant solid ammonium- and iodo-functionalized product (fCNC) was isolated by filtration, washed with water and characterized as described below.

### Physicochemical and structural characterizations of fCNC

High-resolution ultrastructural analyses of unfunctionalized (pristine CNC) and fCNC was performed using a JEOL (Peabody, MA) JEM 1400 Transmission Electron Microscope (TEM). Samples (1 mg/L) were adsorbed onto plasma-treated formvar-coated copper grids. This TEM instrument has accelerating voltage up to 120 kV and is equipped with digital CCD cameras for rapid (2 K × 2 K) or high-resolution (11 Megapixel) imaging. Multiple images of both species were collected, and the dimensions of the particles was measured and tabulated for analysis of characteristic shape, length, and diameter. Atomic force microscopy (AFM) was also performed on pristine CNC and fCNC. Briefly, a stock solution of 0.1 g/L was diluted 1:100, and 0.040 mL of this solution was plated on freshly cleaved mica substrate (SPI) and allowed to stand for 30 min. and then air dried. Molecular Biology Grade H_2_O (Fisher) was used to wash the samples (10 mL) and dried under a gentle stream of nitrogen gas. Imaging was performed using an MFP-3D-BIO microscope (Asylum Research) equipped with an Olympus AC240TS-R3 AFM probe (Asylum Research) and operated in tapping mode at ambient temperature. Images were captured at a scan size of 2 µm × 2 µm (512 × 512 pixels). AFM images were exported into tiffs from the Asylum Research Igor Pro software. Tagged image file format (Tiff) exported images were then imported into FIJI/ImageJ (NIH) for quantification of height and length of nanomaterials using a custom written FIJI code. Each particle was segmented in FIJI and analyzed separately, with a size filter to remove any debris in the images from the analysis. The top pixel intensity for each particle was used to determine the height of each nanomaterial, and a Feret’s diameter measurement was used to calculate length. All data was analyzed using GraphPad Prism 7 software (GraphPad Software). Size exclusion (SEC) chromatography analyses were performed using a Shimadzu HPLC system comprised of a LC-20AB liquid chromatograph, SIL-20ACHT auto sampler, RF-20XS fluorescence detector, SPD-20AV UV/Vis detector, SPD-M20A diode array detector, and an inline Lab Logic γ-RAM Model 4 radioactivity detector. The raw data obtained from each chromatograph was processed and plotted using GraphPad Prism 7. The stationary phase was a Superdex 200 column (Amersham Biosciences) using a 20 mM sodium acetate/150 mM sodium chloride/0.2% sodium azide mobile phase at a flow rate of 0.5 mL per minute. The amine content of the fCNC was quantified using the Sarin assay as previously described^21^. Briefly, to conduct this quantitative assay, a 1 M glycine (Fisher Scientific) solution was prepared and serially diluted in distilled water to yield glycine concentrations of 5, 10, 25, 50, and 100 nM and measured along with a water blank to construct a standard curve. The glycine solutions were formulated into a volume of 0.100 mL. The fCNC samples were analytically weighed and dissolved in distilled water to prepare solutions of known concentration by mass and similarly prepared in dilutions of 0.100 mL final volume. To each 0.100 mL sample (glycine or fCNC) was added 0.100 mL of absolute ethanol (Sigma Aldrich) and 0.050 mL of each Kaiser test kit (Fluka) reagent: (a) ninhydrin, 6% in ethanol; (b) phenol, 80% in ethanol; and (c) potassium cyanide in water/pyridine. These 0.350 mL solutions were added to a Pyrex test tube and heated for 5–7 min at 102 °C after which time they were added to a cuvette along with 0.80 mL of water and 0.20 mL of absolute ethanol and the absorbance at 570 nm was measured. The mole of amine in each fCNC sample was extrapolated using the plot of absorbance at 570 nm versus mole of amine obtained from the linear plot of the glycine standards. The amine loading of fCNC was reported as mmole of amine per gram of fCNC. Fourier Transform Infrared (FTIR) Spectroscopy was performed using a Brukner Tensor 27 with an ATR platform and OPUS 7.5 software installed. Samples were deposited onto KBr plates and the system was set to ATR mode and spectra were collected at 4 cm^−1^ resolution from 500–4000 cm^−1^. Spectra were collected for unmodified CNC, fCNC and EBEA.

### Tracer labeling of fCNC with radionuclide and fluorophore for studies in vivo

Amine-functionalized CNC was radiotracer labeled as described previously for amine-functionalized carbon nanotubes^15^. Briefly, 37 MBq (1 mCi) of acidic ^225^Ac nitrate (U.S. Department of Energy (ORNL, TN)) in 0.2 M HCL was added to a solution of 1.15 mg of S-2-(4-Isothiocyanatobenzyl)-1,4,7,10-tetraazacyclododecane tetraacetic acid (DOTA-Bz-SCN, Macrocyclics, Inc.) in 0.115 mL water. The pH was adjusted with the addition of 0.1 mL of 2 M tetramethylammonium acetate (Aldrich) and 0.02 mL of 150 g/L l-ascorbic acid (Aldrich) to yield a pH 5.5 radiolabeling reaction mixture. The reaction was heated at 58 °C for 30 min. The fCNC (3 mg in 0.3 mL water) was added to the [^225^Ac]DOTA-Bz-SCN reaction mixture and the pH was raised to 9.5 with the addition of 0.15 mL of 1 M carbonate/bicarbonate buffer solution. The reaction was held at 37 °C for 75 min. and subsequently quenched with 0.020 mL of 50 mM diethylenetriaminepentaacetic acid (DTPA, Aldrich). The reaction mixture was purified by preparative size exclusion chromatography using a P6 resin (BioRad) as the stationary phase and 1% human serum albumin (HSA, Swiss Red Cross) in 0.9% NaCl (Abbott Laboratories) as the mobile phase. An aliquot of the final tracer-labeled product, [^225^Ac]fCNC, was used to determine the radiochemical purity by instant thin layer chromatography using silica gel (ITLC-SG). ^225^Ac activity was assayed in a Squibb CRC-15R Radioisotope Calibrator (E.R. Squibb and Sons, Inc.) set at 775 and multiplying the displayed activity value by 5 at secular equilibrium. fCNC was also tracer-labeled with AlexaFluor 488 using conventional bioconjugation methods as previously described for amine-functionalized carbon nanotubes^15^. Briefly, fCNC was dissolved in a reaction buffer of 0.1 M phosphate, 0.15 M NaCl, pH 7.5 with 20% acetonitrile (v/v). To this reaction mixture was added an amine-reactive AlexaFluor488 tetrafluorophenyl ester (AF488-TFP, Invitrogen). The AF488 was reacted at a ratio of 1 dye per 2 amines at ambient temperature for 2 h, adjusting the pH to 8.5 with 10 N NaOH to hydrolyze any remaining unreacted dye. This mixture was purified by dialysis using 3 kD molecular weight cut-off membrane to yield fCNC-AF488. The AF488 stoichiometric substitution was quantified by UV/Vis spectrophotometry (SpectraMaxM2) as previously described^15^.

### Biodistribution of radiotracer labeled fCNC in rodents

The kinetics of tissue distribution, blood clearance and renal elimination of radiotracer-labeled fCNC was examined in two groups (n = 3 per group) of immunocompetent C57BL6 mice (Jackson Laboratory, 5 months old, female). Each animal received a single intravenous dose of 11.1 kBq (300 nCi) of [^225^Ac]fCNC. Mice were euthanized at 1 and 72 h and tissue samples (blood, heart, lungs, liver, spleen, stomach, small and large intestines, kidneys, muscle, bone, brain and urine) were harvested, weighed and counted in a γ-counter (Packard Instruments) using a 360–480 keV energy window at secular equilibrium (24 h postharvest). Aliquots (0.020 mL) of the injected activity were used as decay correction standards. The percent of the injected activity per gram of tissue weight (%IA/g) was calculated and plotted as means. Statistical analysis of data was performed using GraphPad Prism 7 software. All animal experiments were done in accordance with the NIH guide for the care and use of laboratory animals and approved by the Institutional Animal Care and Use Committee of Memorial Sloan Kettering Cancer Institute.

### Tissue distribution of fluorescent-labeled fCNC in rodents

Five immunocompetent C57BL6 mice (5 months old, female) were examined to identify the cellular distribution of fluorophore-labeled fCNC. Each animal received a single intravenous dose of fCNC-AF488 and after 24 h, the heart, kidneys, liver, and spleen were harvested, washed in ice cold PBS, and fixed overnight in 4% paraformaldehyde at 4 °C. Fixed tissue was subsequently embedded in paraffin, and sectioned to obtain 0.005 mm samples. Immunofluorescent (IF) staining was performed as previously described^17,18^ using a Discovery XT processor (Ventana Medical Systems). Briefly, tissue sections were deparaffinized with EZPrep buffer (Ventana Medical Systems), antigen retrieval was performed with CC1 buffer (Ventana Medical Systems) and sections were blocked for 30 min with Background Buster solution (Innovex). The anti-AF488 antibodies (Molecular Probes, cat. no. A-11094, 5 µg/mL) were applied and sections were incubated for 5 h, followed by 60 min. incubation with biotinylated goat anti-rabbit IgG (Vector labs, cat. no. PK6101, 1:200 dilution)^15,17,18^. Slides were counterstained with 4′,6-diamidino-2-phenylindole, (DAPI, Sigma Aldrich, 5 μg/mL) for 10 min. and coverslipped with Mowiol. The tissue distribution fCNC was imaged using anti-AF488 IF staining. Images were acquired using Pannoramic Flash (3D Histech) using a 20 × 0.8NA objective.

### Toxicity evaluation of fCNC in vitro

The toxicity of fCNC was assayed in vitro using human kidney epithelial cells. Briefly, HEK293T cells (293 T, ATCC, cat. no. CRL-3216) were incubated with varying concentrations of fCNC. HEK293T cells were seeded at 1 × 10^4^ per well in a 96-well plate with 0.10 mL DMEM media. The next day the media was removed, and the cells were treated with 0.10 mL of serial dilutions of fCNC ranging from 0.39 μg per well to 50 μg per well and 0.1 M HCl, all in 0.10 mL DMEM. After incubation for 48 h, the media was removed, and the cell viability was assayed with CellTiter-Glo (Promega). Cell viability did not decrease across with the range of fCNC doses. The 0.1 M HCl control was included as a positive indication of toxicity and cell death. Each treatment group was replicated in triplicate.

### Toxicity evaluation of fCNC in vivo

The toxicity of fCNC in vivo was assayed in naïve, healthy mice that were injected intraperitoneally (IP) with fCNC and their body weight was measured as the primary readout for toxicity. Briefly, C57Bl/6 mice (4–6 months old) were injected IP with either 200 μL PBS vehicle, 200 μL PBS containing 1.5 mg or 15 mg of fCNC on day 0. The mice were observed for lethargy and grooming and weighed every 2–3 days for 2-weeks post-injection.

## Results and discussion

Two key chemical modifications were implemented to derivatize each hydroxyl moiety present on pristine high order (crystalline) CNC in order to isolate an amine-functionalized product that is more water-soluble than the starting crystalline nanocellulose material. The synthetic scheme introduced iodide and amine groups on the cellulose surface (Fig. 1). Our strategy included an initial conversion of the secondary hydroxyl groups on the C2 and C3 ring carbons to an iodide substituent. The goal of this step was to reduce hydroxyl content thereby decreasing intermolecular hydrogen bonding and improving molecular dispersion and aqueous solubility. In a following reaction, primary hydroxyls on the C5 carbon were reacted with epichlorohydrin, thus introducing a reactive epoxide that could be opened with 2,2′-(ethylenedioxy)bis(ethylamine) via nucleophilic attack by a diamine under basic conditions. Sarin assay found 0.285 mmol of amine per gram of fCNC that equals 51.3 mmol of amine per mole glucose. Therefore, there is approximately 1 amine per 19.5 glucose units, but note that many of the glucose units are buried inside the particle. The resulting product is an ammonium- and iodo-functionalized product (fCNC) with reactive primary amines and improved water solubility. Further chemical modifications to introduce radionuclide and fluorescent tracer-moieties yielded [^225^Ac]fCNC (96% radiochemically pure and 0.2% radiochemical yield) and fCNC-AF488 (> 95% pure and 33% chemical yield). Spectroscopic and Kaiser assay analysis of the fCNC-AF488 indicated that there was 1 AF488 appended per 30 glucose units.

**Figure 2:**
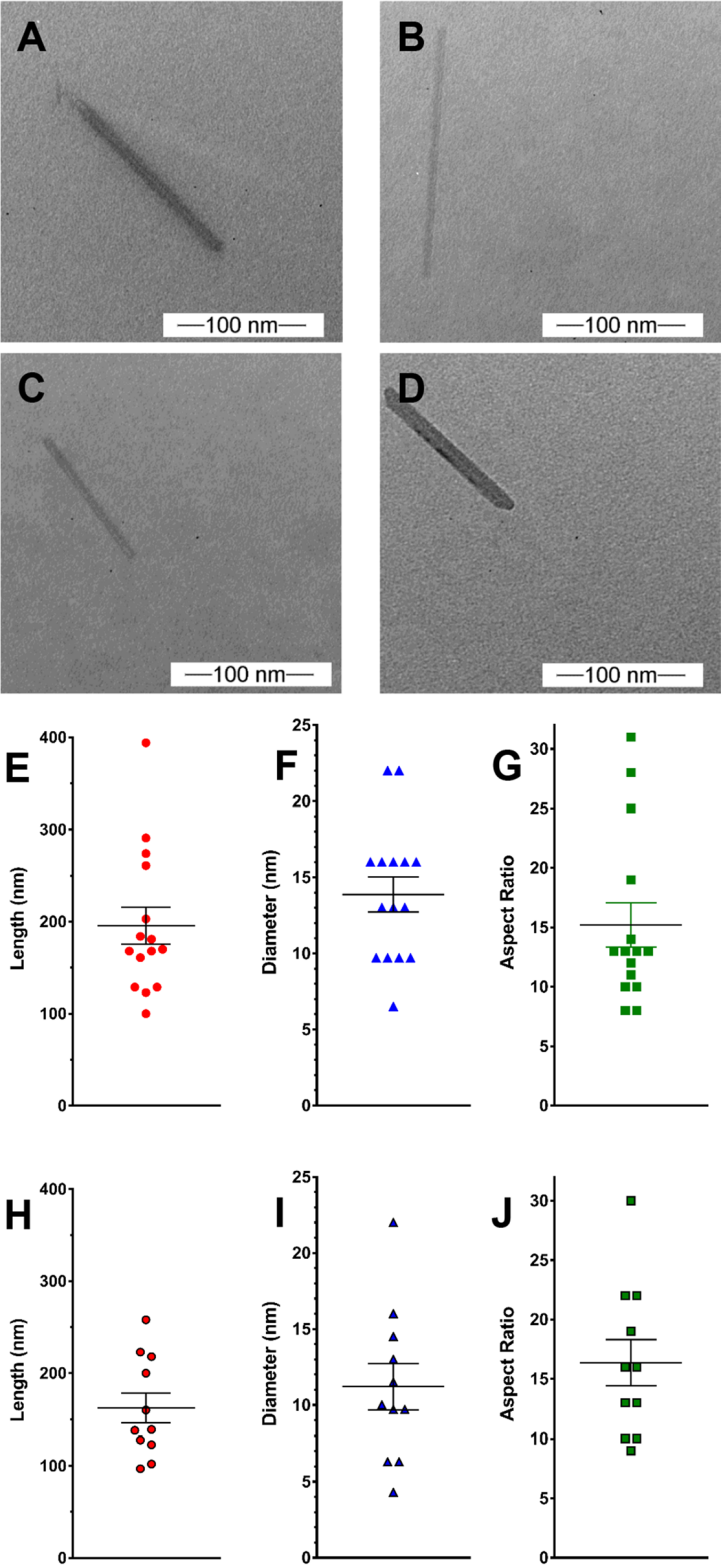
Representative TEM images of pristine CNC (**A,B**) and fCNC (**C,D**) show linear molecular shapes for both the starting material and the product. The characteristic high aspect ratio and rigid linear shape of the starting material is preserved following the functionalization procedures. Analysis of pristine CNC particle length (**E**), diameter (**F**), and aspect ratio (**G**) shows a length of 195.7 ± 20.2 nm, diameter of 13.9 ± 1.16 nm, and aspect ratio of 15.2 ± 1.87 (mean ± SEM, n = 15). Analysis of fCNC particle length (**H**), diameter (**I**), and aspect ratio (**J**) shows a length of 162.4 ± 16.3 nm, diameter of 11.2 ± 1.52 nm, and aspect ratio of 16.4 ± 1.94 (mean ± SEM, n = 11).

The structural characteristics (length, diameter, and aspect ratio) of pristine CNC and fCNC were measured using TEM and AFM. Representative TEM images of pristine CNC (Fig. 2A,B) and fCNC (Fig. 2C,D) show quite linear rod-like molecular shapes for both the starting material and the product. The characteristic high aspect ratio and rigid linear shape of the starting material is preserved following the functionalization procedures. Analysis of pristine CNC particle length (Fig. 2E), diameter (Fig. 2F), and aspect ratio (Fig. 2G) shows a length of 195.7 ± 20.2 nm, diameter of 13.9 ± 1.16 nm, and aspect ratio of 15.2 ± 1.87 (mean ± SEM, n = 15). Analysis of fCNC particle length (Fig. 2H), diameter (Fig. 2I), and aspect ratio (Fig. 2J) shows a length of 162.4 ± 16.3 nm, diameter of 11.2 ± 1.52 nm, and aspect ratio of 16.36 ± 1.94 (mean ± SEM, n = 11). Interestingly, the particle diameters were observed to cluster at 6, 10, 13, 16, and 22 nm displaying incremental unit increases of approximately 3 nm suggesting that uniform fiber bundling persists from the original molecules.

**Figure 3:**
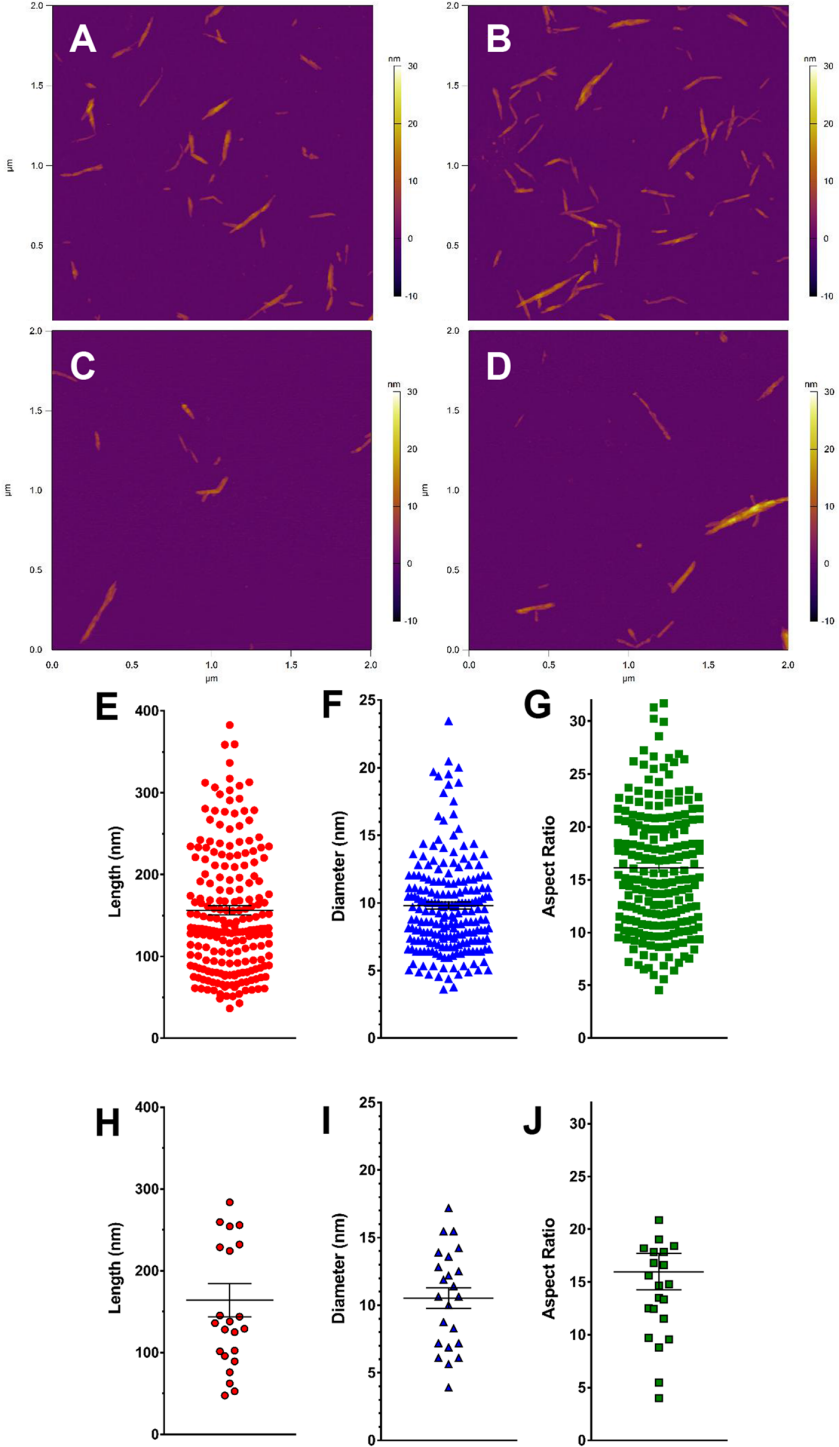
Representative AFM images of pristine CNC (**A,B**) and fCNC (**C,D**) on a mica substrate show linear shaped molecules in both the starting material and the product populations. The characteristic high aspect ratio and rigid linear shape of the starting material is preserved following the functionalization procedures and confirms the TEM results. Analysis of pristine CNC particle length (**E**), diameter (**F**), and aspect ratio (**G**) shows a length of 156.6 ± 5.70 nm, diameter of 9.81 ± 0.27 nm, and aspect ratio of 16.14 ± 0.43 (mean ± SEM, n = 214). Analysis of fCNC particle length (**H**), diameter (**I**), and aspect ratio (**J**) shows a length of 164.0 ± 20.3 nm, diameter of 10.52 ± 0.76 nm, and aspect ratio of 15.97 ± 1.73 (mean ± SEM, n = 23).

AFM measurements corroborated the TEM findings confirming the linear macromolecular shape of the raw material (Fig. 3A,B) as well as the fCNC (Fig. 3C,D). We calculate that based on these numbers there are approximately nine fibrils per particle, each with approximately 160 glucose units per fibril. Analysis of pristine CNC particle length (Fig. 3E), diameter (Fig. 3F), and aspect ratio (Fig. 3G) shows a length of 156.6 ± 5.70 nm, diameter of 9.81 ± 0.27 nm, and aspect ratio of 16.14 ± 0.43 (mean ± SEM, n = 214). Analysis of fCNC particle length (Fig. 3H), diameter (Fig. 3I), and aspect ratio (Fig. 3J) shows a length of 164.0 ± 20.3 nm, diameter of 10.52 ± 0.76 nm, and aspect ratio of 15.97 ± 1.73 (mean ± SEM, n = 23). We estimate from our data that the molecular weight of a fCNC with mean length and diameter is approximately 260,000 g/mole and is comprised of up to nine fibrils assuming a 3.5 nm fibril diameter^5^.

**Figure 4:**
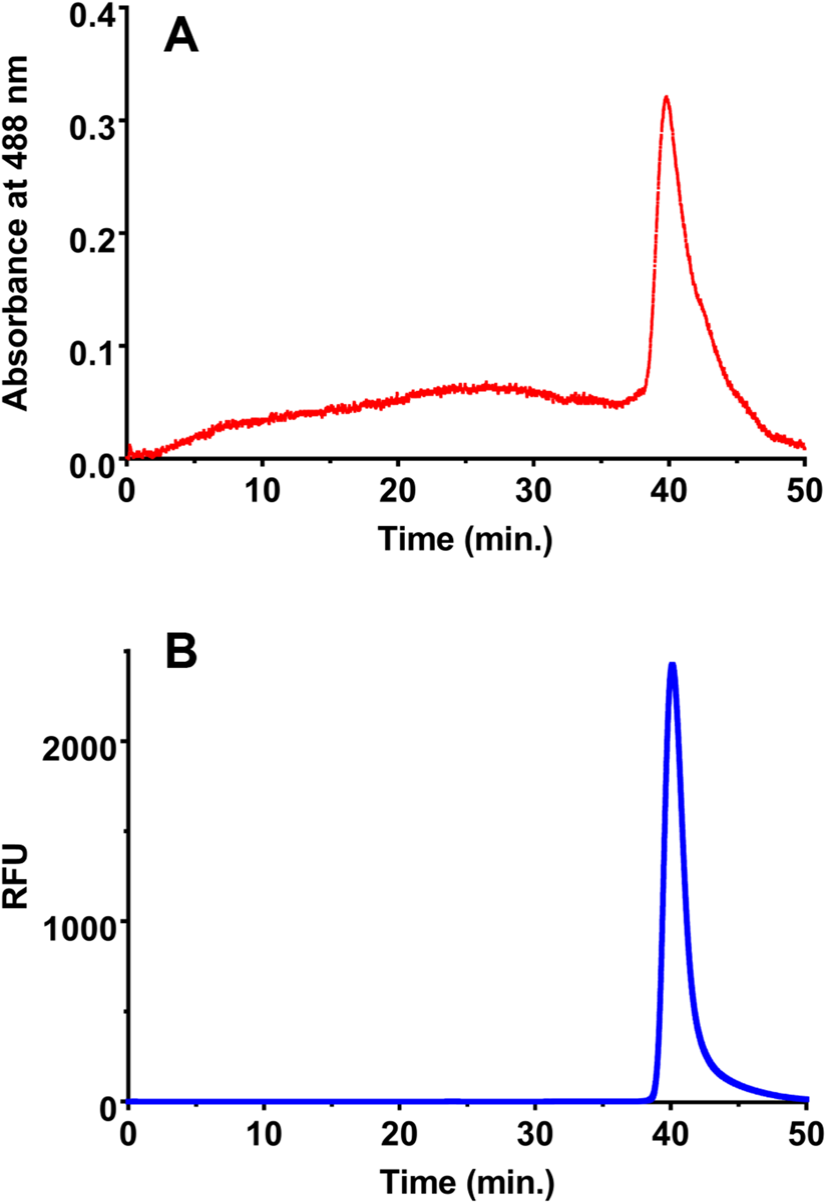
Size exclusion chromatography data of fCNC in aqueous mobile phase. (**A**) UV–Vis absorbance trace at 488 nm and (**B**) fluorescence (excitation at 495 nm and emission at 519 nm) trace of fCNC-AF488.

Chromatographic analysis of an aqueous solution of the linear, high molecular weight fCNC-AF488 using analytical size exclusion HPLC demonstrated the water solubility of the product. The distinctive high aspect ratio of this linear shaped macromolecule resulted in a delayed elution more typical of a globular-shaped molecule that is two-to-three orders of magnitude smaller in molecular weight^15^. The elution profile of the absorbance trace at 488 nm (Fig. 4A) establishes that high molecular weight fCNC-AF488 (approximately 260,000 g/mole) elutes at 41 min., which is in the regime where small molecules typically elute under these stationary and mobile phase conditions. The corresponding fCNC-AF488 fluorescence trace (Fig. 4B) correlates with the fCNC absorbance trace in Fig. 4A. We have demonstrated previously that functionalized carbon nanotubes (fCNT) that are of similarly high aspect ratio dimensions relative to this novel functionalized nanocellulose product also elute in the small molecular weight region of the chromatograph^20^. The water-soluble amine-functionalized carbon nanotubes and cellulose nanocrystals materials are both linear macromolecules and both of these functionalized molecules align with the mobile phase flow and are eluted with late retention times from the stationary phase under similar conditions.

FTIR spectroscopy did not identify amine peaks in the fCNC (Supplemental Fig. 1). The spectra of the EBEA diamine shows a weak N–H stretch at 3376 cm^−1^ and a weak amide peak at 1596 cm^−1^. The spectra of fCNC’s does not display either of these two peaks, presumably due to the broad hydroxyl peak at ~ 3400 cm^−1^ and the peak at 1596 cm^−1^ was not observed. Pristine CNC and fCNC display a characteristic -OH group feature ~ 3400 cm^−1^; a D-glycosyl peak at 892 cm^−1^; a C-O stretch at 1032 cm^−1^; and C–O–C stretches at 1160 cm^−1^.

**Figure 5:**
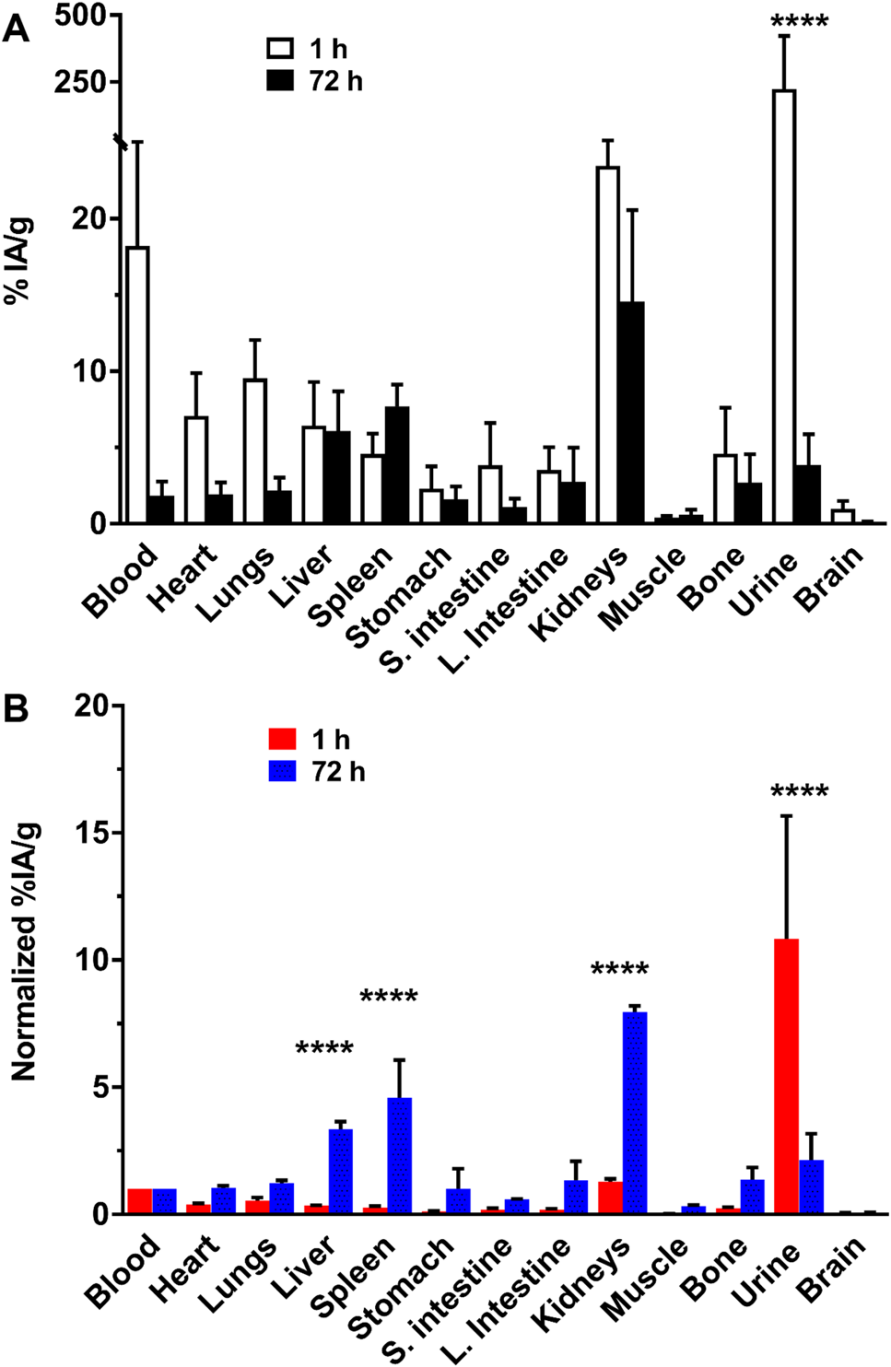
Biodistribution and clearance of [^225^Ac]fCNC in immunocompetent mice at 1 and 72 h post intravenous administration showing tissue biodistribution, blood clearance and renal elimination. (**A**) The percent of the injected activity per gram (%IA/g) of [^225^Ac]fCNC in each sample and (**B**) the %IA/g normalized to blood activity showing the specific accumulation of fCNC in liver, spleen, and kidney along with elimination into the urine.

The interface of fCNC with biological systems was investigated in an immunocompetent mouse model. Figure 5A reports the tissue biodistribution and blood and urine clearance of [^225^Ac]fCNC in mice and is reported as the %IA/g. Importantly, these data establish rapid clearance of intravenously administered [^225^Ac]fCNC from the blood compartment with significant prompt renal elimination into urine (*P* < 0.0001). Normalization of the tissue %IA/g results (mean ± SD) to blood activity highlights the accumulation of activity after 72 h in kidneys (7.7 ± 0.1), liver (3.4 ± 0.9), and spleen (4.6 ± 2.1) (Fig. 5B). The accumulation of [^225^Ac]fCNC in kidneys (*P* < 0.0001), liver (*P* < 0.0001) and spleen (*P* = 0.0004) was significantly increased after 3 days relative to blood and the amount clearing in the urine was decreased at that time (*P* < 0.0001). Importantly, little uptake of [^225^Ac]fCNC is noted in the heart, lungs, stomach, intestines, muscle, bone and brain. We have described a similar nanomaterial pharmacokinetic profile previously for fCNT and attribute the uptake of [^225^Ac]fCNC in these three tissues based upon their biological roles in processing and clearance of linear polymeric solutes in the blood^12–20^. The fibrillar pharmacokinetic behavior of this novel cellulose-based nanomaterial has been revealed to provide similar blood and renal clearance as well as analogous tissue accumulation when compared to fCNT. Indeed, when the fCNC was labeled with [^225^Ac]DOTA and administered intravenously in a naïve mouse model we recorded greater than 50% of the injected activity cleared from the blood into the urine within 1 h postinjection. Furthermore, 23%IA/g accumulated in the kidneys within 1 h post intravenous administration and 15%IA/g was present in this organ after 72 h. In comparison, about 10%IA/g of fCNT accumulated in the kidneys after 1 h post-administration, which suggests that the fCNC platform can potentially deliver more drug cargo into the kidneys compared to fCNT.

**Figure 6:**
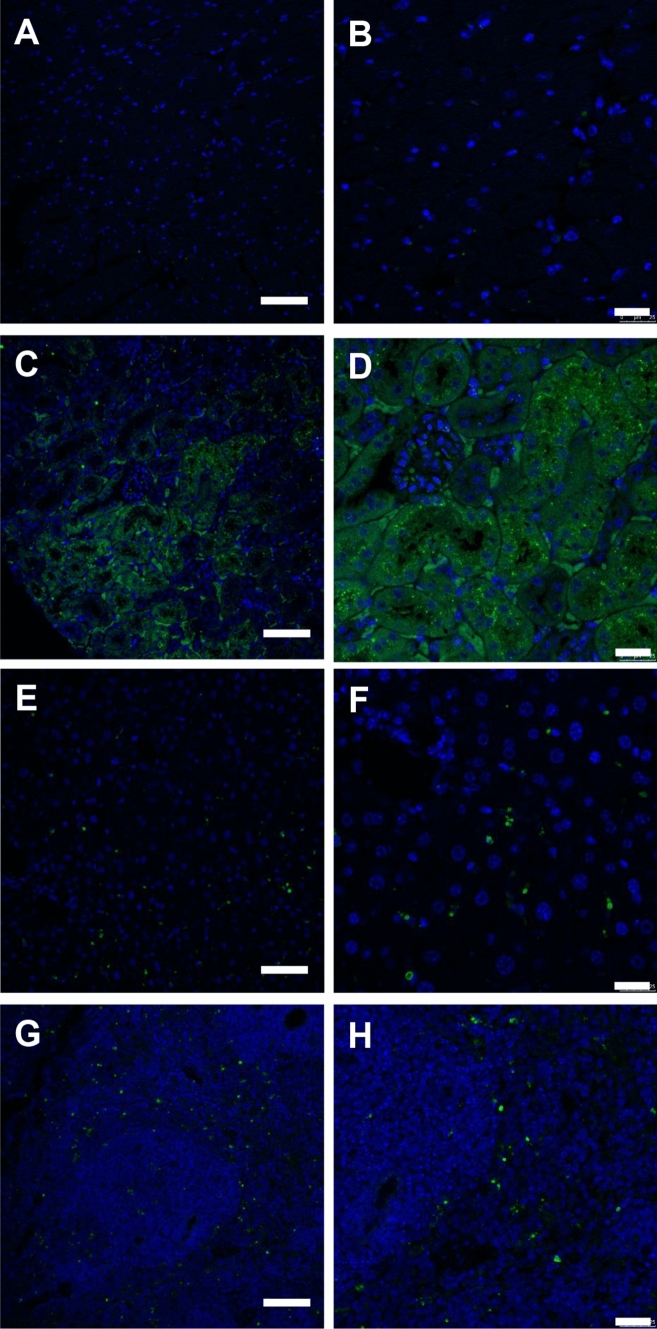
Immunofluorescence images of fCNC-AF488 in mouse **(A,B)** heart, **(C,D)** kidney, **(E,F)** liver and **(G,H)** spleen tissue. Anti-AF488 staining (green) 24 h after intravenous administration of fCNC-AF488. Tissue distribution of nanomaterial was not evidenced in heart tissue but accumulation was noted in kidney, liver and spleen. Scale bars are for Panels (**A,C,E,G)** are 75 µm; scale bars are for Panels (**B,D,F,H)** are 25 µm. Nuclei (blue) are DAPI-stained.

Tissue-specific uptake of fCNC was confirmed using IF imaging of fCNC-AF488 in heart (Fig. 6A,B), kidney (Fig. 6C,D), liver (Fig. 6E,F), and spleen (Fig. 6G,H) sections stained for AlexaFluor488 (green). Nuclei are stained blue with DAPI. Heart was included as control since it does not accumulate fCNC.

**Figure 7:**
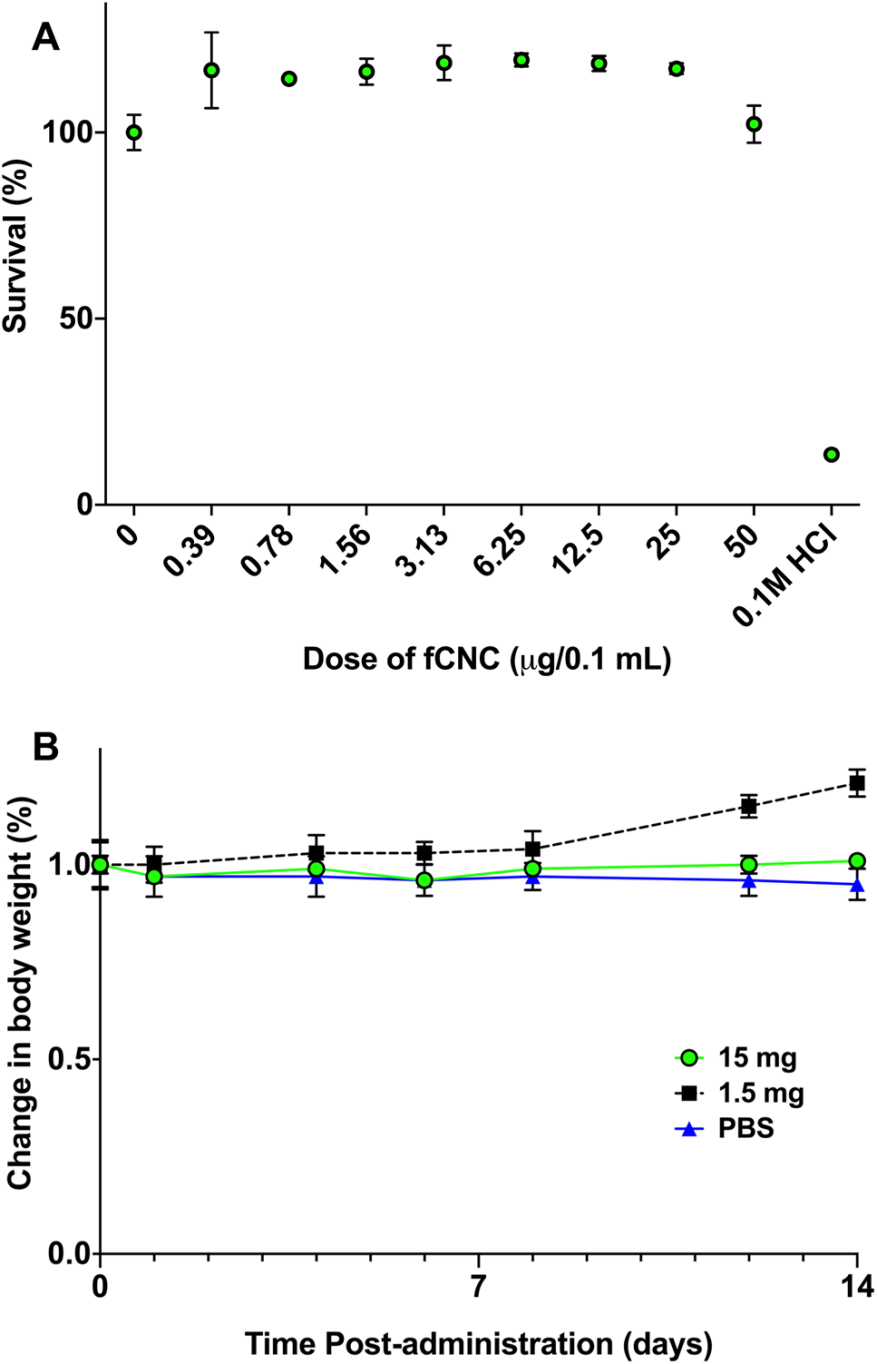
Biocompatibility and safety of fCNC **(A)** in vitro and **(B)** in vivo. There was no evidence of human kidney epithelial cell toxicity arising from treatment with fCNC in vitro. Cell viability did not decrease across a range of fCNC doses. The fCNC was biocompatible and safe in naïve mice as indicated by absence of weight loss upon treatment. There was no signs of lethargy or absence of grooming noted in these animals.

The novel, functionalized fCNC was safe in vitro and in vivo. There was no evidence of human kidney epithelial cell toxicity arising from treatment with fCNC in vitro (Fig. 7A). Cell viability did not decrease across a range of fCNC doses. Nor was there toxicity in vivo in naïve mice as indicated by absence of weight loss upon treatment (Fig. 7B). There was no signs of lethargy or absence of grooming noted in these animals.

## Summary and conclusions

Cellulose nanocrystals are an abundant naturally occurring organic biopolymer resource and we now show water-soluble functionalized derivatives exhibit unique fibrillar nanomaterial properties in vivo. Technologies utilizing safe, inexpensive and biocompatible nanomaterial must be developed to harness their properties for appropriate biomedical applications as excipients in relevant diagnostic and therapeutic applications. Research investigating natural polymers^1^ has increased in recent years as evidenced by the number of reports of medical implants, drug delivery systems, antimicrobials, wound dressings, vascular grafts, and scaffolds for tissue engineering^2^. Interestingly, nanocellulose is already widely used as a low-calorie additive to thicken, bind, and carry flavor in a variety of food products (e.g., soups, gravies, fillings, bread, chips, crackers, meat, and pudding) due to exceptional rheological behavior^22–28^.

These linear polysaccharides (molecular weight approximately 260,000 g/mole) display a pharmacological profile in vivo that differs significantly compared to globular shaped molecules^11–15,17–19^. Renal-specific targeting is of particular importance in drug discovery and we have shown that linear macromolecules with high aspect ratio (> 10) align with blood flow, are filtered via the glomerulus, and subsequently reabsorbed in the nephron from the luminal boundary^15^. Ideally, drug cargo that is bound to the nanomaterial excipient is delivered to those tubule cells due to the pharmacokinetic profile of the delivery platform. Our laboratory has been interested in designing fibrillar nanomaterials that are biocompatible and safe and deploying them in drug delivery strategies^12–20^. Previously, we have reported that fCNT is safe and biologically functional as an excipient for drug delivery to the renal cortex^17^. Indeed, our findings reported herein suggest that we can deploy functionalized nanocellulose as an excipient to target and deliver RNAi to the kidneys.

The fCNC biopolymer should be readily accepted for use in vivo given its widespread use in food science as an additive^22–28^. Interestingly, one key difference between the biodistribution profiles of fCNC and fCNT is the greater blood persistence of fCNC at 1 h^14,15^. Another difference is the preferential targeting of the renal medulla cell population versus the renal cortex for fCNT^12–15,17,19^. This difference will offer a fibrillar nanoplatform that clears the blood more slowly than fCNT and is able to accumulate to a greater extent in a different region of the kidney. Major aims of our translational drug delivery program are focused on RNA interference strategies to treat renal injury and disease^16,17^. Few current platforms allow reliable, specific, and protected delivery of siRNA to the kidney in vivo^17,29,30^. The introduction of fCNC as a novel nanomaterial excipient provides a unique nanodelivery platform with characteristic fibrillar pharmacokinetics. Furthermore, the fCNC platform should realistically decrease raw material costs associated with the catalytic synthesis of carbon nanotubes. Moving forward, we continue to investigate different chemical modifications of pristine CNC in order to further modify pharmacokinetics and establish functionalization procedures with siRNA as well as other biological agents.

## Supplementary Information

Supplementary Information.
