# Supplementary material for: Fibrillar pharmacology of functionalized nanocellulose
Source: Sci Rep. 2021 Jan 8;11:157. doi: 10.1038/s41598-020-79592-5 (PMC7794391; doi:10.1038/s41598-020-79592-5)
Supplement: Supplementary file 1 — Supplementary Information. [file 41598_2020_79592_MOESM1_ESM.pdf]

Fibrillar pharmacology of functionalized nanocellulose

Sam Wong[1,2,‡], Simone Alidori[1,‡], Barbara P. Mello[1], Bryan Aristega Almeida[1], David Ulmert[3], Matthew B. Brendel[4], David A. Scheinberg[3,5], and Michael R. McDevitt[1,6,*]

[1] Department of Radiology, Memorial Sloan Kettering Cancer Center, New York, NY 10065.

[2] Department of Chemistry, Hunter College, New York, NY 10065.

[3] Molecular Pharmacology Program, Memorial Sloan Kettering Cancer Center, New York, NY 10065.

[4] Molecular Cytology Core Facility, Memorial Sloan Kettering Cancer Center, New York, NY 10065.

[5] Department of Pharmacology, Weill Cornell Medicine, New York, NY 10065.

[6] Department of Radiology, Weill Cornell Medical College, New York, NY 10065.

[*] Corresponding author: Michael R. McDevitt, Ph.D., Department of Radiology, Memorial Sloan Kettering Cancer Center, 1275 York Avenue, Box 231, New York, New York 10065; (646) 888-2192 Tel.; (646) 422-0640 Fax; mcdevitm@mskcc.org.

[‡] These authors contributed equally to this work.

Supplemental Figure 1. FTIR Spectra of unmodified CNC, fCNC and EBEA.

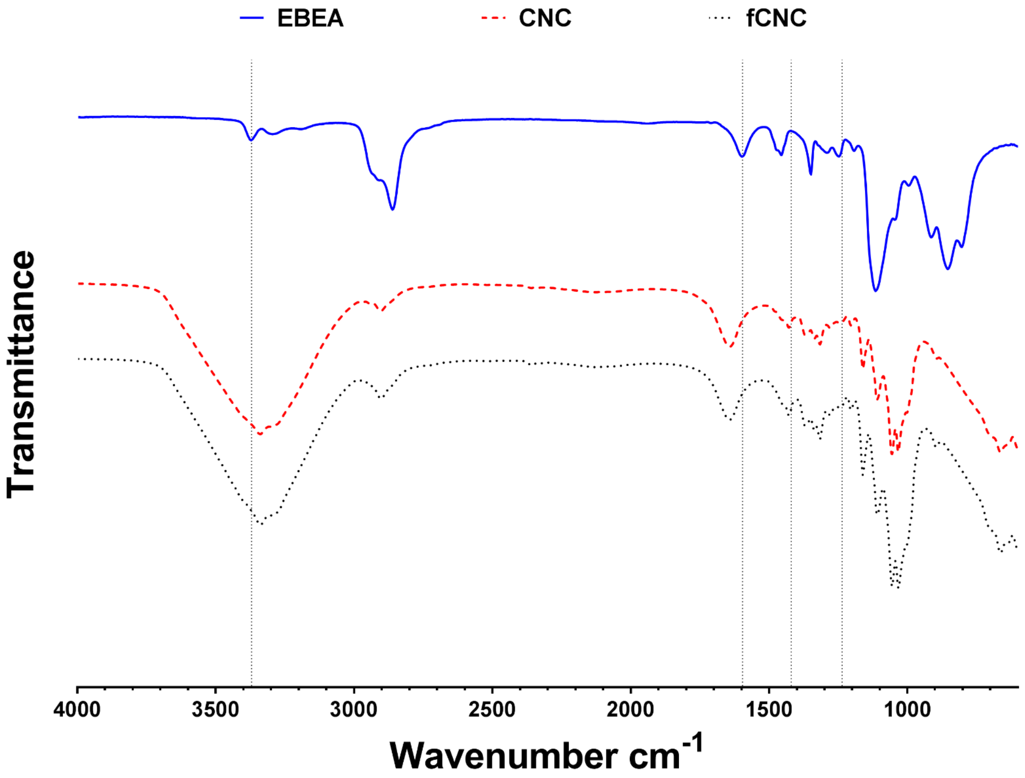

EBEA
CNC
fCNC
Transmittance
4000
3500
3000
2500
2000
1500
1000
Wavenumber cm-1
